# Extreme Peripheral Blood Plasmacytosis Mimicking Plasma Cell Leukemia as a Presenting Feature of Angioimmunoblastic T-Cell Lymphoma (AITL)

**DOI:** 10.3389/fonc.2019.00509

**Published:** 2019-06-13

**Authors:** Kelsey Sokol, Saritha Kartan, William T. Johnson, Onder Alpdogan, Neda Nikbakht, Bradley M. Haverkos, Jerald Gong, Pierluigi Porcu

**Affiliations:** ^1^Department of Medical Oncology, Thomas Jefferson University, Philadelphia, PA, United States; ^2^Department of Dermatology and Cutaneous Biology, Thomas Jefferson University, Philadelphia, PA, United States; ^3^Blood Cancer and BMT, University of Colorado, Aurora, CO, United States; ^4^Department of Pathology, Thomas Jefferson University, Philadelphia, PA, United States

**Keywords:** angioimmunoblastic T-cell lymphoma, plasmacytosis, EBV, plasma cell leukemia, follicular helper T-cell

## Abstract

Angioimmunoblastic T-cell lymphoma (AITL) is one of four major subtypes of nodal peripheral T cell lymphoma, characterized by its cell of origin, the follicular helper T-cell (T_FH_). Patients typically present with prominent constitutional (B) symptoms, generalized lymphadenopathy, hepatosplenomegaly, cytopenias, and rash. Here we present a case of a 62-year-old male with progressive cervical adenopathy, fevers and weight loss presenting with extreme polyclonal plasmacytosis and high plasma EBV viral load. While the initial presentation appeared to mimic plasma cell leukemia or severe infection, lymph node biopsy and bone marrow biopsy confirmed a diagnosis of AITL. This case highlights the heterogeneity of the clinical presentation of AITL to enable physicians to more promptly recognize, diagnose and initiate treatment.

## Introduction

Angioimmunoblastic T cell lymphoma (AITL) is recognized in the 2016 World Health Organization (WHO) Classification as one of the four major subtypes of nodal peripheral T cell lymphoma (PTCL), accounting for 15–25% of all PTCL, depending on geographical region, and approximately 1–2% of all non-Hodgkin lymphomas (NHL) (1). AITL is a clinically distinct lymphoid neoplasm, with a well-characterized cell of origin, the follicular helper T-cell (T_FH_), and with discrete immunophenotypical and genetic features. Most patients with AITL present with very prominent constitutional (B) symptoms, generalized lymphadenopathy, hepatosplenomegaly, multilineage cytopenias (in particular thrombocytopenia), and rash (2). These symptoms may precede the diagnosis by several months, rarely by years. Laboratory signs of B-cell activation and autoimmunity are a hallmark of AITL, including hypergammaglobulinemia, autoimmune hemolytic anemia (AIHA), immune mediated thrombocytopenia (ITP), and presence of autoantibodies (rheumatoid factor, anti-phospholipid, anti-DNA, and anti-smooth muscle antibodies). The majority of AITL patients have detectable Epstein Bar Virus (EBV)-encoded RNA (EBER)-positive cells by *in situ* hybridization (ISH) (3). in the diagnostic lymph node biopsy and about 50% have measurable plasma EBV DNA (pEBVd) (4), even before initiation of chemotherapy which may contribute to polyclonal B-cell activation and lead to the development of EBV-positive B-cell lymphomas in a subset of patients. While in most cases the hypergammaglobulinemia is polyclonal, monoclonal immunoglobulins (Ig) can be detected in the serum of some patients, mimicking multiple myeloma (5).

There are several reported cases of marked peripheral blood and/or bone marrow plasmacytosis in AITL, mimicking plasma cell leukemia, especially when associated with elevated Ig levels. The polyclonal nature of the plasmacytosis, however, ultimately suggests a reactive etiology, such a viral infection (EBV, parvovirus B19, hepatitis), autoimmune disease (rheumatoid arthritis, systemic lupus erythematosus, Sjögren's syndrome), or serum sickness (6, 7). EBV has an established association with numerous B-cell and T cell lymphoproliferative neoplasms, including AITL (8, 9). Here we report the case of a patient with AITL who presented with marked peripheral blood polyclonal plasmacytosis, polyclonal hypergammaglobulinemia, and high levels of pEBVd, suggestive of EBV reactivation. The case highlights the heterogeneity of the clinical manifestations of AITL, to enable physicians to more readily recognize the disease at presentation and initiate treatment promptly.

## Case Presentation

A 62-year-old male, with a history of non-ischemic cardiomyopathy presented with new onset atrial flutter. He reported shortness of breath, diaphoresis and lightheadedness for ~ 1 week as well as progressive cervical adenopathy, fevers, and unintentional weight loss. Laboratory evaluation demonstrated white blood cell (WBC) count of 17.3 ×10^9^/L with 37% (6.4 ×10^9^/L) plasma cells, hemoglobin of 11.6 g/dL, and platelet count of 53 ×10^9^/L. Contrast-enhanced computed tomography (CT) scans of the chest, abdomen, and pelvis revealed non-bulky cervical, axillary, mediastinal, retroperitoneal and inguinal lymphadenopathy, ranging in size between 1.4 cm and 2.5 cm, as well as splenomegaly of 15.5 cm, without discrete lesions. Shortly after admission, the patient developed acute renal failure and serum electrolyte abnormalities consistent with tumor lysis syndrome (TLS) (creatinine of 4.2 mg/dL, potassium of 5.4 mmol/L, phosphate of 5.3 mg/dL, urate of 11.9 mg/dL, lactate dehydrogenase of 368 IU/L). Peripheral blood flow cytometry revealed that 46% of the leukocytes were represented by polyclonal plasma cells (CD19+, CD20−, CD22−, CD45+(moderate), CD38+(bright), CD56−, CD117−, CD138+, HLA-DR+(heterogeneous), surface κ or λ−, cytoplasmic κ +(subset), cytoplasmic λ+(subset)). Serum protein electrophoresis (SPEP) and immunofixation electrophoresis (IFE) showed abnormally high gamma globulin levels (IgA 1200 mg/dL and IgG 4200 mg/dL) without a monoclonal paraprotein. Serum kappa and lambda light chain levels were modestly elevated (92.6 mg/dL and 73 mg/dL, respectively) but the ratio was normal (1.27).

There was initial concern for an infectious etiology for the patient's presentation given the signs of systemic inflammatory response syndrome (tachycardia, leukocytosis, fever) and circulating polyclonal plasma cells. Blood cultures and viral studies were performed and were notable for a detectable plasma EBV viral load, which was quantified by qRT-PCR as 71,000 copies/mL of pEBVd, and approximately 24 h later increased to 1.05 ×10^6^ copies/mL. Human immunodeficiency virus (HIV) and hepatitis C serologies were negative, hepatitis B serology was consistent with prior immunization, and plasma cytomegalovirus DNA (pCMVd) was negative. Serum antinuclear antibody (ANA) titer for initial workup of autoimmune disease was also negative. After bone marrow and lymph node biopsies were obtained, dexamethasone 40 mg daily was initiated, as concerns for an EBV associated lymphoproliferative disorder (EBV-LPD) were high. In addition, the patient was started on antiviral therapy with ganciclovir 2.5 mg/kg IV every 12 h for possible active EBV infection. The drug was stopped after 5 days as the treating physicians concluded that the risk of drug toxicity outweighed the benefit, and the data to support its use in this setting were insufficient. Despite these measures and aggressive supportive care, within 1 week of admission, his clinical course progressed to multiorgan failure requiring mechanical ventilation and ultimately cardiac arrest requiring extracorporeal membrane oxygenation (ECMO).

**Figure 1:**
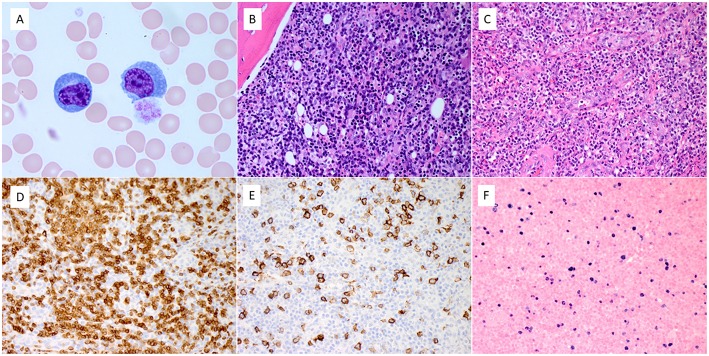
Pathologic features of blood, bone marrow, and lymph node. **(A)** The blood smear showed a marked increase of reactive circulating plasma cells. **(B)** Bone marrow biopsy showed diffuse infiltration of atypical lymphocytes admixed with numerous reactive plasma cells. **(C)** The left inguinal lymph node revealed diffuse infiltration of small to medium-sized atypical lymphocytes and scattered large immunoblasts, with exuberant vascular proliferation and numerous reactive plasma cells. **(D–F)**. The atypical lymphocytes expressed CD3 **(D)**. Scattered immunoblasts expressed CD20 **(E)** and EBER by in situ hybridization **(F)** (Magnification: **A**. x100, **B–F**. x40).

Bone marrow exam revealed hypercellularity (90%) with 30–40% plasma cells (which was deemed to be reactive because of polyclonal kappa/lambda expression), large lymphoid aggregates with predominance of T cells, and scattered EBV+ B cells. Molecular studies for T-cell receptor (TCR) gamma gene and immunoglobulin heavy chain (IGH) gene rearrangements were performed on the blood sample. A monoclonal TCR gamma gene rearrangement was detected; clonal IGH gene rearrangement was not detected. The left inguinal node showed completely effaced normal lymph node architecture with a diffuse infiltration of atypical small to medium sized lymphocytes in a background of vascular proliferation, plasma cells, and scattered immunoblasts. By immunohistochemistry and flow cytometry, there was a diffusely increased T cells infiltrate admixed with scattered B immunoblasts. The T cells expressed CD2, CD3, CD5, CD7, PD-1, and were negative for CD5, CD10, BCL-6, and all B-cell antigens analyzed. CD4 to CD8 ratio was within normal range. Scattered large immunoblasts were positive for CD20 and EBV (by EBER *in situ* hybridization). CD21 revealed several expanded clusters of follicular dendritic cells. *In situ* hybridization for immunoglobulin kappa and lambda light chains revealed numerous polyclonal plasma cells. Molecular studies for TCR gamma gene and IGH gene rearrangements were repeated on the lymph node. Identical clonal TCR gamma gene rearrangement was detected in the lymph node. Similarly, clonal IGH gene rearrangement was not detected. Representative images are shown in Figure 1.

In aggregate, these findings were thought to be most consistent with a diagnosis of AITL. Pathologically, the complete effacement of nodal architecture, diffuse involvement of bone marrow, and the presence of clonal T-cell gene rearrangement rule out conditions of reactive EBV infection such as infectious mononucleosis and chronic active EBV infection. Unfortunately, the patient failed to improve with dexamethasone and the family decided to withdraw care. A request for autopsy was declined.

## Discussion

In this report we present a case of AITL with profound polyclonal peripheral plasmacytosis and rapidly rising pEBVd, reaching extremely high levels (>1 million copies/mL). This presentation offered a significant challenge as plasma cell leukemia and a severe systemic infection were the initial diagnostic considerations.

AITL is an aggressive subtype of nodal peripheral T cell lymphomas. It is derived from follicular helper T-cells (T_FH_) that normally reside at the border between the mantle zone and the germinal center of the normal physiologic lymph node (10). T_FH_ are a distinct subset of T helper cells necessary for the formation of germinal centers (GC) and B-cell differentiation. They are formed through naïve CD4+ T-cell's interaction with dendritic cells, leading to the activation of the inducible T-cell costimulator (ICOS) and phosphatidylinositol 3-kinase (PI3K) pathways in the CD4+ T-cells. This induces the expression of B-cell lymphoma 6 protein (BCL6), along with other transcription factors which guides the differentiation of the naïve CD4+ T-cells into T_FH_ (10, 11) T_FH_ then help in directing the differentiation of B-cell centroblasts to centrocytes, which ultimately form plasma cells or memory B cells (10). T_FH_ begin to express high levels of C-X-C chemokine receptor type 5 (CXCR5), PD-1, BCL6, myelin-associated glycoprotein (MAG), and serum amyloid P component (SAP), the expression of which can be detected by immunohistochemistry (IHC). The genetic alterations thought to be driving the development of AITL include ten eleven translocation methylcytosine dioxygenase 2 (TET2), DNA (cytosine-5)-methyltransferase 3A (DNMT3A), isocitrate dehydrogenase (NADP(+)) 2 (IDH2), ras homolog gene family member A (RHOA), and components of the TCR pathways, all of which are potentials targets for therapy (11).

Histologic examination of involved lymph nodes in AITL shows small-medium sized tumor cells in small clusters with clear cytoplasm, that can be identified as T_FH_ by immunohistochemistry, based on the expression of PD-1 and CXCL13 (CXCR5 ligand) and pan T-cell markers, such as CD3 and CD4 (12). The associated FDC are typically identified by expression of CD21, CD23, and CD35. These two cell types are present among a population of other small reactive lymphocytes, histiocytes, and plasma cells, with high endothelial venules (13). The B-cells are often EBV+ (EBER+), while the associated T cells and plasma cells are typically not (14). This morphologic appearance can initially be mistaken for a hyperplastic reactive lymph node with EBV reactivation.

In a retrospective subset analysis of 157 patients with AITL enrolled on the GELA (Groupe d'Etude des Lymphomes de l'Adulte) LNH87 and LNH93 randomized clinical trials, bone marrow involvement was seen in 60% of patients (14). The most commonly seen features were paratrabecular and interstitial infiltration of atypical lymphocytes, histiocytes and eosinophils, with infiltration of small to large B cells. Vascular proliferation and reticulin fibrosis have also been reported. Secondary changes include trilineage hyperplasia and polyclonal plasmacytosis. Together, these findings can lead to misdiagnoses, including benign lymphoid hyperplasia, T cell rich large B cell lymphoma, chronic myeloproliferative disease, or plasma cell dyscrasia (15–17). The consensus is that CD10, CXCL13, and PD-1 are expressed in only a fraction of cases, although double labeling with CD3 and BCL6 may specifically highlight the neoplastic T cells (16). Additionally, depending on the methodology used, T-cell receptor (TCR) gene rearrangements are seen in 70–90% of cases and Ig gene rearrangements in approximately 10-20% of cases (18).

The association between polyclonal plasma cell proliferations and AITL has not yet been fully elucidated. While it may in part be secondary to cytokine release such as IL-6, in this case we hypothesize that the EBV likely was a contributing factor (6). EBV reactivation is known to occur when latently infected B cells are exposed to a variety of stimuli, including physiologic signals able to induce proliferation and differentiation (19). The specific mechanisms leading to the occurrence of high levels of pEBVd in AITL merit further investigation.

We report this clinical case study to emphasize the need for consideration of AITL in patients presenting with peripheral blood polyclonal plasmacytosis, high levels of pEBVd, and features highly suggestive of lymphoma, such as extensive lymphadenopathy and TLS. While the initial presentation and tissue morphology appears consistent with a reactive process, closer examination as above reveals histopathology consistent with AITL. In previous reports of AITL with plasmacytosis, patients who received rapid treatment showed a good response to cytotoxic chemotherapy (20). The efficacy of treatment in patients with AITL and significant EBV viral load remains less well known.

## Data Availability

No datasets were generated or analyzed for this study.

## Patient Consent

Written consent for publication of this case report and any potentially identifying information was obtained by the patient's wife.

## Author Contributions

KS and PP wrote the first draft of the manuscript. SK, WJ, OA, NN, BH, JG, and PP wrote sections of the manuscript. All authors contributed to manuscript revision, read and approved the submitted version.

### Conflict of Interest Statement

The authors declare that the research was conducted in the absence of any commercial or financial relationships that could be construed as a potential conflict of interest.
